# A97 PHYSICIAN AND PATIENT PERCEPTIONS REGARDING LOW DOSE ASPIRIN FOR THE PREVENTION OF PREECLAMPSIA IN PREGNANT PEOPLE WITH INFLAMMATORY BOWEL DISEASE

**DOI:** 10.1093/jcag/gwae059.097

**Published:** 2025-02-10

**Authors:** H Hothi, R Jogendran, Y Hanna, V Srikanth, K O’Connor, P Tandon, V Huang

**Affiliations:** Medicine, University of Toronto, Toronto, ON, Canada; Medicine, University of Toronto, Toronto, ON, Canada; Medicine, University of Toronto, Toronto, ON, Canada; Medicine, University of Toronto, Toronto, ON, Canada; Medicine, University of Toronto, Toronto, ON, Canada; Medicine, University of Toronto, Toronto, ON, Canada; Medicine, University of Toronto, Toronto, ON, Canada

## Abstract

**Background:**

Preeclampsia, characterized as high blood pressure and end organ dysfunction in pregnancy, is the second leading cause of maternal mortality worldwide. Patients with inflammatory bowel disease (IBD) are at increased risk of developing severe preeclampsia. While there is emerging evidence that prevention with low dose aspirin (LDA) is safe in pregnant patients with IBD, patient and physician perceptions of LDA use remain unclear.

**Aims:**

To examine Canadian patient and physician perceptions on LDA use for the prevention of preeclampsia in pregnant people with IBD.

**Methods:**

This is a cross-sectional study with a survey for patients with IBD and a second survey for Canadian healthcare providers (HCPs). We collected demographic information and Likert scale answers on perceptions of LDA use including perceived side effects, utility, and optimal prescribing practices. Bivariate assessments were performed using Chi-square analysis and Fischer’s Exact Test.

**Results:**

125 patients and 46 HCPs responded. Patient median age was 35 and 60.8% were diagnosed with Crohn’s disease, 40% with Ulcerative Colitis, and 2.4% with indeterminate disease. 73.15% of patients did not consider Aspirin to pose significant risks such as increased gastrointestinal side effects, IBD flares, or fetal harm. 64.8% of patients agreed with being comfortable taking aspirin if prescribed for preeclampsia risk. There was no association between age, education level, income, or ethnicity and perceived comfort (all p > 0.05). Among HCPs, 43% were gastroenterologists, 30% obstetricians, and 27% other (General Practitioners, General Internists, Nurse Practitioner, Rheumatologist, Midwife, Gynecologist). Respondents (38.0%) most commonly recommended that obstetricians should prescribe LDA. For dosages, 51% of HCPs indicated 162 mg daily and 16.3% indicated 81 mg daily should be prescribed if indicated, while 18.4% reported not knowing. There was no association between years in practice, setting (academic versus community),or volume of IBD or pregnant patients seen in clinic (i.e., scope of practice), with HCP reported comfort levels in managing pregnancy related care in IBD (all p > 0.05). Scope of practice was also not associated with HCP perception that pregnant patients with IBD would develop pre-eclampsia or have worse outcomes than those without IBD.

**Conclusions:**

Although IBD patient and HCP perceptions vary, LDA use is generally accepted for pre-eclampsia prophylaxis in pregnant patients with IBD. Future research should focus on targeted educational approaches to increase awareness and uptake of LDA therapy.

**Funding Agencies:**

CAG

